# Supplementary material for: Learning curve and surgical outcome of robotic assisted colorectal surgery with ERAS program
Source: Sci Rep. 2022 Nov 29;12:20566. doi: 10.1038/s41598-022-24665-w (PMC9709162; doi:10.1038/s41598-022-24665-w)
Supplement: Supplementary file 2 — Supplementary Information 2. [file 41598_2022_24665_MOESM2_ESM.zip › RAL Raw data-Ñ[▒K/102/OP N_ñw╝╨░O▒Kñσ.pdf]

W83 / W83

臺中榮民總醫院
手術室護理記錄

[redacted]

基本資料

- 室別： 開刀房-17室，手術科別： CRS，手術類別： 常規，日期： 2021/07/29
- 進入時間： 0730 ； 麻醉開始時間： 0810 ； Time Out時間： 0900 ； 劃刀時間： 0901 ； 手術結束時間： 1455 送出時間： 1550 ；
- 麻醉方式： GE

  診斷： Malignant tumor of rectum

  手術名稱： 1.Robot Assisted Low Anterior Resection2.Ureter, Ureterolithotomy - U/3 or L/3

  手術局部用藥： nil

  術前預防性抗生素：
    - 抗生素名稱一： Veterin 1GM IVD/由麻醉護理人員給予
    - 時間： 0845
    - 上次簽章: 歐真勤

  ，追加預防性抗生素：
    - 抗生素名稱一： Veterin 1GM IVD/由麻醉護理人員給予
    - 時間： 1245
    - 上次簽章: 蘇敬涵
- 手術醫師： 蔣鋒帆，楊曙榮，陳怡豪
- 麻醉醫師： VS 齊光彪
-
    - 刷手護士A ： 蘇敬涵/廖娸?，時間： 0730，刷手護士B ： 王明盈，時間： 0730，接替刷手一： 王明盈/廖娸?/李芩瑜，時間： 1130，接替刷手二： 侯玟君，時間： 1500
    - 流動護士A ： 歐真勤，時間： 0730，接替流動一： 蘇敬涵，時間： 1200，接替流動二： 林貝臻，時間： 1500

皮膚狀況

A-V Shunt： 無

術前： 完整

記錄者： 上次簽章: 歐真勤

術後： 不完整

部位(術後)：

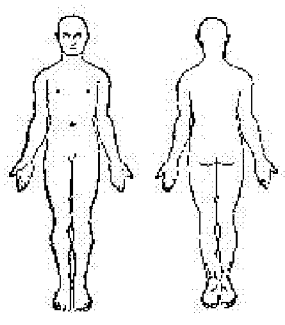

左鼻0.5*0.5CM壓紅不可白（鼻胃管造成）

記錄者： 上次簽章: 林貝臻

手術準備

手術臥式 單一手術臥式

-
    - 手術臥式： 截石臥式
    - 使用附件： 溫毯 38 ℃，矽膠枕頭，腳架
    - 皮膚準備： 10%Aq Betadine，Hibiscrub，2% Chlorhexidine
    - 電燒： 使用電燒: 電極片位置： 其它： 迴路電極墊，記錄者： 上次簽章: 蘇敬涵

止血帶T： 無

植入物

無

標本送檢

- 細菌培養 無
- 細胞學 無
- 冰凍切片 無
- 病理標本
  組織名稱一： IMA root
  ,
  組織名稱二： distal cut-end
  ,
  組織名稱三： rectum
- 截肢 無

標本交付 標本： 3 袋 , 標本： 0 盒 , 標本： 0 瓶 , 細胞學： 0 管 , Culture Tube： 0 管 , 被交付醫師： CR 楊曙榮 ,

- 記錄者： 上次簽章: 蘇敬涵
- 時間： 1310

傷口術後敷料

傷口敷料： 傷口敷料： 無

- 請於圖上標示部位：

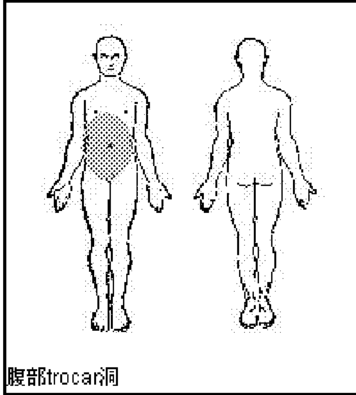

- 傷口敷料： mepore

導管

導管	天數	導管資訊	插入日期	插入時間	更換日期	拔除日期	拔除時間	拔除原因	預計拔除/更換	預計拔除/更換日期
Foley(1)	1	號碼:16 Fr.; 部位:膀胱;	2021/07/29	0830						
Epidural PCA(1)	1	部位:硬脊膜下;	2021/07/29	0815						
A-line (1)	1	號碼:20 G; 部位:左手;	2021/07/29	0815						
Penrose Drain(1)	1	號碼:10 mm.; 部位:右側後腹腔;	2021/07/29	1455						
Double-J(1)	1	號碼:6Fr. x 24cm ; 部位:右側輸尿管;	2021/07/29	1455						
Double Lumen CVC(1)	1	號碼:8 Fr.; 深度:15cm; 部位:jugular (右); 象限:RU;	2021/07/29	0825						

用物計數

- 手術中出血量： Minimal c.c.
- 手術中尿量： 1150 c.c.
-
  - 其它： 無

用物計數： 多切口同時縫合

- 1.器械： 正確，2.縫針： 正確，3.刀片： 正確，4.紗布： 正確，5.腹部墊： 未使用，6.紗球： 未使用，7.PEANUTS ： 未使用，8.紗條： 未使用，9.絲帶： 正確，10.針頭： 未使用，11.Vessel loop： 正確，12.Boots： 未使用，13.Jelco： 未使用，14.棉條： 未使用，
    - 第一次：
        - 刷手護士 ： 王明盈
        - 流動護士： 上次簽章: 蘇敬涵
        - 時間： 1430

      ,
    - 第二次：
        - 刷手護士 ： 王明盈
        - 流動護士： 上次簽章: 蘇敬涵
        - 時間： 1440

      ,
    - 第三次：
        - 刷手護士 ： 王明盈
        - 流動護士： 上次簽章: 蘇敬涵
        - 時間： 1450

備註

備註：davinci 30scopy:NO1

- 記錄者： 上次簽章: 歐真勤
- 時間： 0730

填寫護理人員 林貝臻

正式摘要

W83 / W83

doctorRank:B
